# MRI features of combined hepatocellular- cholangiocarcinoma versus mass forming intrahepatic cholangiocarcinoma

**DOI:** 10.1186/s40644-018-0142-z

**Published:** 2018-02-27

**Authors:** Jennifer Sammon, Sandra Fischer, Ravi Menezes, Hooman Hosseini-Nik, Sara Lewis, Bachir Taouli, Kartik Jhaveri

**Affiliations:** 1 Toronto Joint Department of Medical Imaging, University Health Network, Sinai Health System and Women’s College Hospitals, University of Toronto, Toronto, Canada; 2 Department of Pathology, University Health Network, University of Toronto, Toronto, Canada; 3 Department of Radiology, Mount Sinai New York, New York, USA

**Keywords:** Combined hepatocellular-cholangiocarcinoma, Intrahepatic cholangiocarcinoma, Biphenotypic tumor, Liver MRI, Primary liver tumor

## Abstract

**Background:**

Combined hepatocellular-cholangiocarcinoma (cHCC-CC) is a rare primary liver tumor, which has overlapping imaging features with mass forming intra-hepatic cholangiocarcinoma (ICC) and hepatocellular carcinoma (HCC). Previous studies reported imaging features more closely resemble ICC and the aim of our study was to examine the differential MRI features of cHCC-CC and ICC with emphasis on enhancement pattern observations of gadolinium enhanced MRI.

**Methods:**

Institutional review board approval with consent waiver was obtained for this retrospective bi-centric study. Thirty-three patients with pathologically proven cHCC-CC and thirty-eight patients with pathologically proven ICC, who had pre-operative MRI, were identified. MRI images were analyzed for tumor location and size, T1 and T2 signal characteristics, the presence/absence of: cirrhosis, intra-lesional fat, hemorrhage/hemosiderin, scar, capsular retraction, tumor thrombus, biliary dilatation, degree of arterial enhancement, enhancement pattern, pseudocapsule and washout. Associations between MRI features and tumor type were examined using the Fisher’s exact and chi-square tests.

**Results:**

Strong arterial phase enhancement and the presence of: washout, washout and progression, intra-lesional fat and hemorrhage were all strongly associated with cHCC-CC (*P* < 0.001). While cHCC-CC had a varied enhancement pattern, the two most common enhancement patterns were peripheral persistent (*n* = 6) and heterogeneous hyperenhancement with washout (*n* = 6), compared to ICC where the most common enhancement patterns were peripheral hypoenhancement with progression (*n* = 18) followed by heterogeneous hypoenhancement with progression (*n* = 14) (*P* < 0.001).

**Conclusion:**

The cHCC-CC enhancement pattern seems to more closely resemble HCC with the degree of arterial hyperenhancement and the presence of washout being valuable in differentiating cHCC-CC from ICC. However the presence of washout and progression, in the same lesion or a predominantly peripheral /rim hyperenhancing mass were also seen as important features that should alert the radiologist to the possibility of a cHCC-CC.

## Background

Combined hepatocellular-cholangiocarcinoma (cHCC-CC) is a rare primary liver tumor that expresses both biliary and hepatocellular markers on immunohistochemistry. The WHO reclassified cHCC-CC in 2010 into two subgroups: cHCC-CC classical type and cHCC-CC with stem cell features. These tumors must show unequivocal hepatocellular (HCC) and cholangiocarcinoma (ICC) components which have transition zones, thus differentiating cHCC-CC from collision tumors [1].

As cHCC-CC is a rare tumor, only a few studies have looked at prognosis and management of this tumor, with complete tumor resection and lymph node clearance having the best prognosis. Survival rates post resection appear to be worse than HCC and similar to ICC [2–7], with several studies reporting 5-year survival rates of 16–41.1% for cHCC-CC post-transplant compared to near 70% for HCC patients [8–11]. There are no accepted transplant criteria for cHCC-CC to date, with previous studies reporting poor outcome post liver transplant for patients with presumed HCC who were found to have cHCC-CC on the explant pathology. As patients can proceed to transplant without histology, pre-operative diagnosis of cHCC-CC is important, but remains challenging, as there is both clinical and radiological overlap in these tumors. cHCC-CC can occur in patients with risk factors for HCC and in patients with risk factors for ICC and due to the heterogeneity of the tumor, cHCC-CC can have overlapping imaging features with HCC and ICC. Tumor markers cannot be relied upon to differentiate, as only just over half of patients in one study had elevated Alpha-fetoprotein (AFP) and/or carbohydrate antigen 19.9 (CA19.9) [1].

Previous studies report imaging features of cHCC-CC appear to more closely resemble ICC and metastasis rather than HCC [12–18] and to the best of our knowledge there are only a few studies that have attempted to investigate the MRI features of cHCC-CC [12–15]. We performed a step-wise systematic evaluation of MRI examinations of pathologically proven cHCC-CC versus ICC. The aim of our study was to examine the differential MRI features of cHCC-CC and ICC with emphasis on enhancement pattern observations of gadolinium enhanced MRI.

## Methods

### Patients

Institutional review board approval with consent waiver was obtained for this retrospective bi-centric study. Pathology databases at both centers were searched for consecutive cHCC-CC/biphenotypic tumors between January 2005 and December 2014 and these results were cross-referenced with radiology databases, excluding any patients who did not have preoperative MRI. Over the same period the pathology and radiology databases were searched for ICC cases.

The patient demographics of the two groups are summarized in Table 1. Thirty-three patients who had pathologically proven cHCC-CC and MRI at baseline were identified. Within this cohort, 25 of the patients were male and 8 were female. The mean age was 59.5 years with an age range of 36–82. Twenty-five patients had chronic liver disease: 16 patients had hepatitis B, 9 patients had hepatitis C, 3 patients had a history of alcohol abuse, 1 patient had hemochromatosis, 1 patient had non-alcoholic steatohepatitis and 1 patient had primary biliary cirrhosis. Two of the patients with histories of alcohol excess were also hepatitis C positive and 1 patient had both hepatitis B and hepatitis C positive serology. Twenty-three (69.7%) of the patients had cirrhosis on imaging, defined as lobar redistribution (hypertrophy of the caudate and left lateral segments, with atrophy of the right lobe and left medial segments) and/or nodular hepatic contour.

**Table 1:** Patient demographics

Parameter	cHCC-CC	Cholangiocarcinoma
Mean age (range)	59.5 (36–82)	61 (32–86)
Sex (M:F)	25:8	20:18
Median AFP (range)	23.5 ng/ml (< 5–353,014)	2 ng/ml (< 5–15)
Median Ca19.9 (range)	25 U/ml (< 1–49)	16.5 U/ml (< 1–129,207)
Hepatitis B	16	7
Hepatitis C	9	3

AFP was recorded for 29 patients pre-treatment and 8 patients had an AFP > 100 ng/ml, with 5 patients in the cohort having an AFP > 400 ng/ml (range < 5–353,014). Only 7 patients had CA19.9 recorded pre-treatment and 4 of those had elevated CA19.9 (> 37 U/ml), with only one greater than twice the normal limit at 125 U/ml. The remaining patients with a positive CA19.9 ranged from 38 to 49 U/ml.

Forty consecutive patients with pathologically proven ICC with MRI at baseline were identified. Two patients were excluded; one as they did not have dynamic contrast enhanced imaging and the other, as the quality of the study was deemed non-diagnostic. Within this cohort there were a similar amount of male and female patients with 20 males and 18 females. The mean age was 61, with an age range of 32–86. Ten patients had risk factors for liver disease, 7 had hepatitis B and 3 had hepatitis C.

AFP was recorded in 24 patients pre-treatment and no patient had an elevated AFP. CA19.9 was recorded in 26 patients pre-treatment and the median CA19.9 was 16.5 U/ml (range < 1–129,207). Seven patients had a CA19.9 > 37 U/ml.

### Image acquisition

MRI examinations were performed at 1.5 T or 3 T (*n* = 63 at 1.5 T and *n* = 8 at 3 T) using a phased array torso coil. MRI protocol included: T2 single shot turbo spin echo with TE 180, axial T2 turbo spin echo with TE 90, axial T1 volumetric interpolated breath-hold (VIBE) opposed-in phase sequences, axial diffusion weighted imaging and axial T1 VIBE pre-contrast and dynamic post-contrast images (Table 2). The majority of the patients (22 cHCC-CC and 38 ICC) received routine extracellular gadolinium based contrast agent gadobutrol (Gadovist, Bayer Healthcare, Berlin, Germany) at a dose of 0.1 mmol/kg at 1 ml/s. Eleven cases in the cHCC-CC group and 3 cases in the ICC group had imaging with hepatocyte specific contrast agent gadoxetic acid (Primovist, Bayer AG, Germany) at a dose of 0.025 mmol/kg at 1 ml/s. At our institution, the primary contrast agent for initial liver imaging is an extracellular based gadolinium contrast agent, and as this is a retrospective study, only the extracellular phases of contrast imaging were analyzed.

**Table 2:** MRI parameters

Image sequence	TR (ms)	TE (ms)	NEX	FOV (mm)	ST (mm)	Gap (mm)	Matrix (phase x frequency)
Pre-contrast imaging:							
Axial T2 HASTE SPAIR	1600	90	1	360	5	1	259 × 320
Axial T2 HASTE SPAIR	1600	180	1	360	5	1	259 × 320
Axial T1 VIBE opp/in	4.43	1.39–2.49	1	360	3	0	218 × 320
ep2d diff b100,600	7600	66	6	380	5	0	156 × 192
T1 VIBE axial SPAIR	4.19	1.47	1	300	3	0	195 × 320
Post-contrast imaging:							
T1 VIBE axial SPAIR dynamic: arterial (care bolus trigger), venous (45–60 s) and interstitial phase (90–120 s)	4.19	1.47	1	300	3	0	195 × 320
T1 VIBE axial SPAIR 5-min delay	4.19	1.47	1	300	3	0	195 × 320
^a^Post-contrast Primovist:							
T1 VIBE axial SPAIR 20 min	4.37	1.47	1	300	4	0	195 × 320
T1 VIBE axial SPAIR 20 min	4.19	1.47	1	300	1.5	0	202 × 320

^a^If hepatocyte specific contrast agent (gadoxetic acid) used

### Image analysis

Two abdominal radiologists (one abdominal imaging fellow and one faculty with 15 years subspecialty MRI experience) retrospectively reviewed the studies in consensus. Images were reviewed on a picture archive communication system. The following characteristics were evaluated: tumor location and size, T1 and T2 signal characteristics, the presence/absence of: cirrhosis on imaging, intra-lesional fat, hemorrhage/hemosiderin, scar, capsular retraction, tumor thrombus, biliary dilatation, degree of arterial enhancement, enhancement pattern on arterial portal-venous and delayed (5 min) phases, pseudocapsule and washout. T2 intermediate signal intensity was defined as the same signal intensity as the spleen and T2 hyperintense lesions were defined as being of higher signal intensity than the spleen. Capsular retraction was recorded for peripheral tumors, which we defined as being within 1 cm of the liver capsule. The degree of arterial enhancement was defined as being strong if any part of the lesion showed similar enhancement to the aorta, mild to moderate if the enhancement was less than the aorta and absent if there was no arterial enhancement. For the overall enhancement pattern, lesions were characterized as being associated with washout even if there was an area of progressive enhancement in the same lesion as our main aim of this study was comparing cHCC-CC to ICC. Lesions with both washout and progression were captured separately. Lesions were defined as having peripheral enhancement patterns, rather than heterogeneous enhancement patterns, if there was peripheral (< 1 cm depth) enhancement on the arterial or venous phase (in lesions that were hypoenhancing on arterial phase). If there was any central enhancement these lesions were characterized as a heterogeneous enhancement pattern. Evidence of cirrhosis included a lobulated/nodular contour and/or volume redistribution to the left lobe and caudate.

### Statistical analysis

Descriptive statistics (frequencies, percentage, mean) were used to summarize demographics, clinical history and MRI features, by tumor type. Associations between MRI features and tumor type were examined using the Fisher’s exact and chi-square tests. All tests were two sided, and *p* < 0.05 was considered an indicator of a statistically significant association. Statistical analyses were performed using SPSS software (version 20.0, IBM).

## Results

The MRI features of cHCC-CC and ICC are summarized in Table 3. On T1 the majority of the lesions were homogenously hypointense in the cHCC-CC and ICC groups, 23/33 and 30/38 respectively. On T2, the majority of the cHCC-CC group (23/33), had a homogenous intermediate/hyperintense appearance. In the ICC group, 14/38 had heterogeneous signal intensity on T2, 12/38 had homogenous intermediate/hyperintense appearance and peripheral hyperintensity with a central hypointense region was seen in 9/38.

**Table 3:** MRI characteristics of cHCC-CC and ICC

Parameter	cHCC-CC	Cholangiocarcinoma	*P*-value
T1 WI			
Hypointense	23	30	0.37
Heterogeneous	9	5	0.136
Isointense/not seen	1	3	0.375
T2 WI			
Homogenously intermediate/hyperintense	23	12	0.001
Peripheral hyperintensity and central hypointensity	3	9	0.102
Heterogeneous	7	14	0.15
Isointense/not seen	0	3	0.99
Intralesional fat	2	0	0.124
Intralesional hemorrhage	4	1^a^	0.119
Capsular retraction^b^	3/23 (13%)	13/21 (62%)	< 0.001
Cirrhosis on imaging	23	0	< 0.001
Biliary dilatation	5	23	< 0.001
Tumor thrombus	3	0	0.058

^a^This patient had recently had a percutaneous biopsy

^b^Recorded for lesions within 1 cm of the liver capsule

Two patients in the cHCC-CC group had intra-lesional fat and 4 patients in the cHCC-CC group had intra-lesional hemorrhage. No patient in the ICC cohort had intra-lesional fat. One patient in the ICC cohort had evidence of intra-lesional hemorrhage, however this patient had a percutaneous biopsy three days prior to the MRI. Excluding the post biopsy patient in the ICC group, both intra-lesional fat and intra-lesional hemorrhage are highly specific (100%) for cHCC-CC versus ICC, although they have poor sensitivities (6% {95% CI: -2 to 14%} and 12% {95% CI: 1–23%} respectively).

In the cases of peripherally located tumors, 13/21 in the ICC group showed capsular retraction compared to 3/23 in the cHCC-CC group (*P* < 0.001).

The presence of biliary dilatation associated with the mass was seen in 5 of the cHCC-CC group and 23 of the ICC group, *P*-value of less than 0.001. Portal vein tumor thrombus was seen in 3 of the cHCC-CC group compared to 0 in the ICC group.

The enhancement characteristics of cHCC-CC and ICC are summarized in Table 4. Arterial enhancement was seen in 90.9% (*n* = 30) of the cHCC-CC group compared to 57.9% (*n* = 22) of the ICC group. The degree of arterial enhancement in 15 patients in the cHCC-CC group was similar to the degree of enhancement of the aorta (strong) and in the remaining 15 patients it was less intense (mild to moderate) than the aorta, compared to 1 and 22, respectively in the ICC group (*P* < 0.001; strong arterial enhancement). Peripheral rim enhancement on the arterial phase was seen in 14 cases in both the cHCC-CC group and the ICC group.

**Table 4:** Enhancement characteristics of cHCC-CC and ICC

Parameter	Combined HCC/CC	Cholangiocarcinoma	*P* value
Degree of arterial enhancement	Strong: 15/33	Strong: 1/38	< 0.001
	Mild: 15/33	Mild: 22/38	0.295
	Hypo: 3/33	Hypo: 15/38	0.003
Peripheral rim arterial enhancement	14 (42%)	14 (37%)	0.631
Progression	13 (39%)	33 (87%)	< 0.001
Washout	13 (39%)	0	< 0.001
Washout and Progression	3 (9%)	0	0.058

**Fig. 1:**
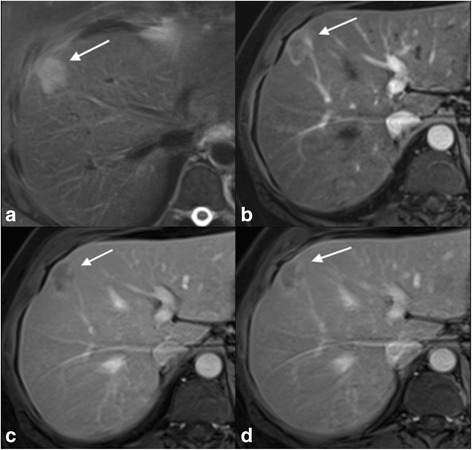
Pathologically proven cHCC-CC with peripheral persistent enhancement: There is a T2 hyperintense (**a**) lesion in segment 8/4A of the liver, which demonstrates peripheral arterial hyperenhancement (**b**). The enhancement pattern remains peripheral on both the portal venous and delayed phases (**c** & **d**). This enhancement pattern (peripheral persistent) was one of the most common enhancement patterns of cHCC-CC seen in our cohort

**Fig. 2:**
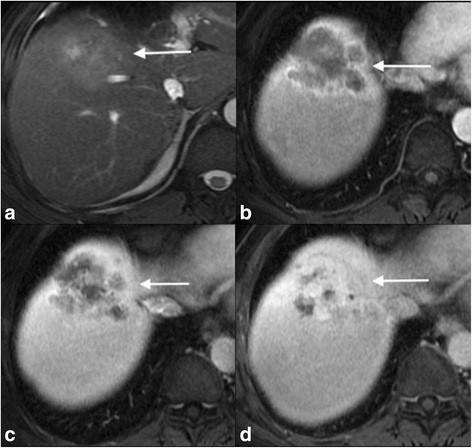
Pathologically proven cHCC-CC with peripheral progressive enhancement: There is a large mass, which is predominantly intermediate signal on T2-WI (**a**) in segment 8 of the liver. This demonstrates peripheral arterial hyperenhancement (**b**), but then shows progressive enhancement on the portal venous and delayed phases (**c** & **d**) demonstrating enhancement pattern similar to that seen in mass forming ICC

With regards to the overall enhancement characteristics of the lesions, the most common enhancement patterns in the cHCC-CC group were peripheral persistent (*n* = 6) (Fig. 1) and heterogeneous hyperenhancement with washout (n = 6). The most common enhancement pattern in the ICC group was peripheral hypoenhancement with progression (*n* = 18) followed by heterogeneous hypoenhancement with progression (*n* = 14) (Fig. 2). Combining peripheral hypoenhancement with progression, heterogeneous hypoenhancement with progression and hypoenhancement versus the other subgroups, there was a statistically significant difference between the ICC and cHCC-CC groups. 79% of the patients who had either one of these three enhancement patterns had ICC and 89% of the patients in the other category had cHCC-CC (*P* < 0.001).

Progressive enhancement was seen in 13 of the cHCC-CC group and 33 of the ICC group (*P* < 0.001). Washout was seen in 13 of the cHCC-CC group and in 0 of the ICC group (P < 0.001), with a sensitivity of 39% (95% CI: 23–56%) and specificity of 100% in differentiating cHCC-CC from ICC. Both washout and progression were seen in the same tumor in 3 cases in the cHCC-CC group.

**Fig. 3:**
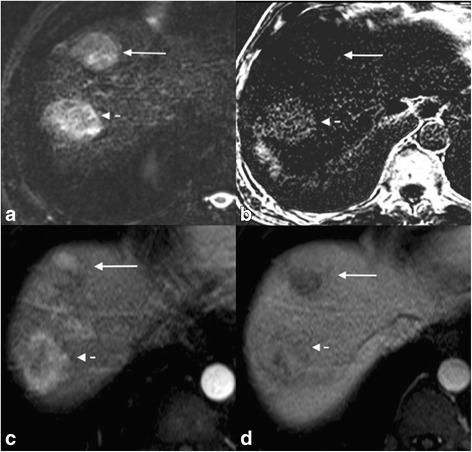
cHCC-CC (long arrow) and HCC (short dashed arrow) in the same liver; show similar T2-WI imaging characteristics (**a**). However the HCC tumor shows intra-lesional fat on in-opposed phased subtraction image (**b**), arterial phase hyper enhancement (**c**) and washout (**d**) compared to the cHCC-CC tumor, which shows no internal fat (**b**) heterogeneous arterial enhancement (**c**) and no washout (**d**). The presence of two different enhancement patterns in similar sized lesions in the same liver should prompt biopsy to confirm that both are HCC as cHCC-CC can occur in the same liver as HCC given the overlap of risk factors

Three patients in the cHCC-CC cohort also had a separate mass characteristic of HCC on their MRI. In two of these cases, the cHCC-CC tumors had similar imaging characteristics to the foci of HCC within the same liver, in that they demonstrated arterial hyperenhancement and washout. In one case, the cHCC-CC and HCC were both over 4 cm in diameter and in this case the cHCC-CC was relatively hypovascular compared to the HCC and it did not contain fat, unlike the HCC. The HCC demonstrated washout, but the cHCC-CC did not (Fig. 3). In two other cases, separate 1–2 cm foci of HCC were identified on the explanted liver, but not detected on pre-operative imaging.

## Discussion

Few studies have been published evaluating the imaging features of cHCC-CC, with most of the earlier studies using the Allen and Lisa or Goodman classifications, which include collision tumors. As mentioned previously most studies report similar imaging characteristics to ICC [12–19]. However, the enhancement characteristics of cHCC-CC in our study appear to more closely resemble HCC rather than ICC, with 13/33 patients in the cHCC-CC cohort having a typical HCC enhancement pattern (arterial enhancement and washout). This may be partly explained by the demographics of our population. In our study the prevalence of cirrhosis (69.7%) and positive hepatitis serology (hepatitis B: 48% and hepatitis C: 27%) in the cHCC-CC cohort is greater than previously reported North American studies [14, 20, 21], where patient demographics and the presence of chronic liver disease risk factors resembled those of ICC rather than HCC. However, some of the earlier studies of cHCC-CC are in Asian populations and these studies report demographics, risk factors and survival similar to HCC [1–3, 22–24]. One European study suggests that the risk factors of the cHCC-CC population lie in between the HCC and ICC groups, but continued to report a male predominance [25]. The differences in our group compared to previously published North American studies could be explained by the increasing Asian population in Canada, higher prevalence of chronic liver disease and increasing incidence of liver cancer [26].

**Fig. 4:**
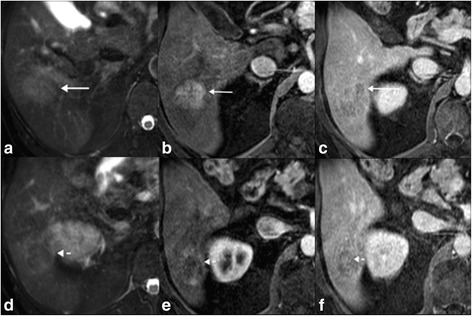
Pathologically proven cHCC-CC tumor demonstrating both washout and progression: **a**-**c** is the superior aspect of the tumor and d-f is the more inferior aspect of the tumor. The superior portion of the tumor is T2 intermediate (**a**) and shows show arterial hyperenhancement and washout (**b**, **c**), typical of HCC. However the more inferior component of the tumor has some internal T2 hypointense components (**d**), and relatively hypovascular on the arterial phase (**e**) and shows some progression of enhancement on the delayed phase (**f**). The presence of washout and progression in the same lesion should alert the radiologist to the possibility of a cHCC-CC tumor

There were also other features associated with HCC in the cHCC-CC group: *n* = 2 had intra-lesional fat and *n* = 4 had intra-lesional hemorrhage. While these features are highly specific in differentiating cHCC-CC from ICC, the low sensitivity does not help in differentiating cHCC-CC from ICC. In 3 cases of cHCC-CC there was both washout and progression in the same lesion (Fig. 4), which does differentiate cHCC-CC from ICC, as washout is not seen in ICC. These features can be seen in scirrhous HCC, but this should alert the radiologist to the possibility of a cHCC-CC tumor and consideration for biopsy as the potential treatment options for these two tumors vary [27, 28].

Previous studies have reported that tumor markers can be helpful in raising the possibility of a cHCC-CC tumor, where both CA19.9 and AFP can be elevated [4, 14, 29, 30]. In our cHCC-CC cohort, AFP was recorded for 29 patients pre-treatment with 8 patients having an AFP > 100 ng/ml and 7 patients had CA19.9 recorded pre-treatment, with 4 of those patients having an elevated CA19.9. While the midrange elevation of AFP helps differentiate these tumors from ICC, there was only one patient who had a CA19.9 above twice the upper limit of normal. This could partially be due to limited sampling in these patients, as the presumptive diagnosis was HCC in the setting of cirrhosis and cHCC-CC was a post resection/explant or post biopsy diagnosis. With previous studies reporting low to mid-level elevation of CA19.9, it does raise an argument for routine CA19.9 testing in patients who have a liver mass as this would alert the radiologist to the possible presence of a cHCC-CC tumor and prompt biopsy, to aid in a pre-treatment diagnosis.

Our study has several limitations, including that this is a retrospective study and the readers were aware the cohort comprised of cHCC-CC and ICC, even though specific pathological diagnosis was not known at the time of image review. Our study group is also small, with only 33 patients in the cHCC-CC group, however this is attributable to the rare nature of this tumor. Despite this, our population for MRI is larger than most other published studies. Another limitation is the absence of histological quantification of HCC and ICC components in the cHCC-CC tumors as not all cases went to resection.

## Conclusion

Pre-operative imaging diagnosis of cHCC-CC tumors remains a challenge. In our study, cHCC-CC tumors displayed predominant arterial hyperenhancement pattern and the presence of washout, similar to HCC, perhaps due to a population with a high prevalence of HCC risk factors. We found that the presence of washout; washout and progression in the same lesion; intra-lesional fat and intra-lesional hemorrhage help differentiate cHCC-CC from ICC.

## Abbreviations

AFP: Alpha-fetoprotein
CA19.9: Carbohydrate antigen 19.9
cHCC-CC: Combined hepatocellular-cholangiocarcinoma
HCC: Hepatocellular carcinoma
ICC: Intra-hepatic cholangiocarcinoma
MRI: Magnetic resonance imaging
